# A Supramolecular Approach to Antimicrobial Surfaces

**DOI:** 10.3390/molecules27175731

**Published:** 2022-09-05

**Authors:** Valentina Gazzola, Pietro Grisoli, Valeria Amendola, Giacomo Dacarro, Carlo Mangano, Piersandro Pallavicini, Antonio Poggi, Silvia Rossi, Barbara Vigani, Angelo Taglietti

**Affiliations:** 1 Department of Chemistry, University of Pavia, Viale Torquato Taramelli 12, 27100 Pavia, Italy; 2 Department of Drug Sciences, University of Pavia, Viale Torquato Taramelli 12, 27100 Pavia, Italy

**Keywords:** copper complexes, microbicidal effect, self-assembled monolayers, surface functionalization, antibacterial surfaces, supramolecular complex, cascade complexes

## Abstract

In this paper, we report on the preparation of Imidazole-functionalized glass surfaces, demonstrating the ability of a dinuclear Cu(II) complex of a macrocyclic ligand to give a “cascade” interaction with the deprotonated forms of grafted imidazole moieties. In this way, we realized a prototypal example of an antimicrobial surface based on a supramolecular approach, obtaining a neat microbicidal effect using low amounts of the described copper complex.

## 1. Introduction

Bacterial and more general microbial infections taking place on surfaces of medical devices, such as dental implants, prostheses, and catheters, are an increasing, serious problem which is spreading worldwide, and which has become dramatic with the recent covid-19 pandemic. Moreover, surface-involving infections can often develop to the form of biofilms, strongly resistant sessile communities of microorganisms embedded in an extracellular polymer matrix produced by the microbes themselves [1]. Biofilms are hard to remove with classical antibiotic treatments, and can lead to the spread of infections, and implants failure and removal, with enormous costs for communities [2].

Anti-infective surfaces based on nanoparticles (NPs) are a modern strategy to try to solve this issue. The toolbox of inorganic nanochemistry offers a wide range of surface modifications, which can render surfaces intrinsically microbicidal. Noble metal nanoparticles, like silver or copper NP, for example, with their high surface/mass ratio, are perfect candidates to be placed on surfaces in the form of grafted monolayers to prevent bacterial adhesion. Metal NPs and released metal ions, in fact, offer several coexisting ways to interfere with bacteria survival and microbial replication, including binding to the cell walls and inflicting damage, generation of reactive oxygen species (ROS) and interaction with DNA, to name a few [3,4,5]. In this way, the generation of bacterial mutations to resist the protection offered is rendered extremely difficult: resistance to silver or copper ions is rare and develops very slowly when compared to resistance to antibiotics which rely on a single mechanism. These kinds of functionalized surfaces are, thus, of strategic importance to prevent infections on cited medical surfaces, but also for coatings intended for shared surfaces of common use, like touch screens, in order to avoid the transmission of infective bacteria and viruses [6].

Grafting of a monolayer of metal complexes on a surface can be another smart tool to counteract bacterial proliferation and biofilm growth, with the obvious advantage of a microbicidal action which can be exerted at very low metal cations concentration values, a precious feature when thinking about the use of potentially toxic elements like silver or copper cations. The use of metal complexes grafted in the form of monolayers on bulk surfaces was, thus, proposed as an alternative to the largely used approach of grafting silver or copper NPs to realize antibacterial surfaces [7]. When considering a monolayer of metal complexes, values of surface concentration found are typically around 2 × 10^14^ units per cm^2^, which corresponds to a few nanograms of metal cations per cm^2^. This value can be compared with the 0.3–0.4 mg of silver per cm^2^ concentration found on a surface which carries a monolayer of silver NPs used for antibacterial tasks [8]. Other advantages of this approach are the cost-effectiveness and the absence of any safety issue related to nano-dimensioned materials. Following this approach, in the recent past, we described the grafting on surfaces of copper (II) complexes of a tetraazamacrocycle [9], 2,2′-bipyridine [10], PEI (polyethyleneimine) [11] and bistren ligands [12,13], the diamino–diamido ligand dioxo-232 [14], and of a silver-sulfadiazine complex [15], demonstrating the efficiency of the union of “classic” coordination chemistry to surface chemistry when pursuing the goal of preparing efficient antibacterial surfaces. On the basis of what was presented in the previous lines of research, in this work we report on the preliminary investigation of a completely novel approach to antimicrobial surfaces, which is based on supramolecular chemistry and metal–ligand interactions.

Our investigation relies on the copper(II) complex of ligand 3,6,9,16,19,22-hexaazatricyclo [22.2.2.2(11.14)]triaconta-1(26),11(12),13,24,27, 29-hexaene, (BPXD), a macrocycle which has been well known for a long time for its ability to coordinate two Cu^2+^ cations [16,17]. Dinucleating copper complexes like [Cu_2_(BPXD)]^4+^ show, in neutral or basic environments, a well-known tendency to bind anions or an imidazolate fragment as a bridging group between two copper cations, a classical example of a supramolecular “cascade complex”, as introduced by Lehn [18]. This concept was investigated several times by the group of Fabbrizzi and co-workers in their quest for receptors and sensing devices for anions [19,20,21,22].

**Scheme 1:**
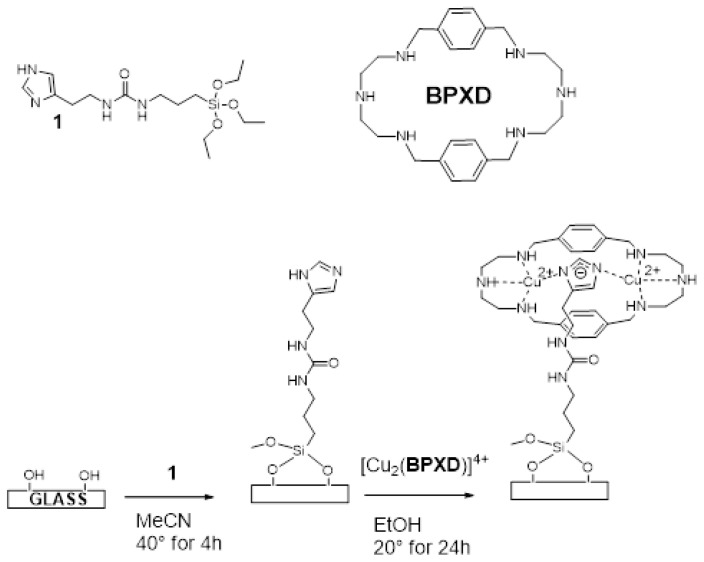
The strategy pursued to bring [Cu_2_(BPXD)]^4+^ on bulk surfaces.

We decided to bind the [Cu_2_(BPXD)]^4+^ complex reversibly to surfaces properly functionalized with a layer of imidazole functions, brought on the surface by exploiting a properly chosen trialcoxysilane with an imidazole fragment appended, exploiting simple alcoxysilane chemistry [6,7,8,9,10,11,12,13,14,15], as depicted in Scheme 1.

The imidazole residue grafted to glass is ready to participate in a cascade complex with [Cu_2_(BPXD)]^4+^, allowing the complex to be established on a bulk surface by means of one of the most powerful non covalent bonds, owing to the toolbox of supramolecular chemistry, the metal–ligand interaction. We describe the solution behavior of [Cu_2_(BPXD)]^4+^ complex in the absence of, and in the presence of, imidazole, the synthesis of the grafting fragment **1**, the functionalization of glass samples with **1** to yield imidazole-functionalized glass surfaces, and their ability to give supramolecular coordinative interaction with [Cu_2_(BPXD)]^4+^. Finally, we demonstrate the powerful antibacterial effects of these functionalized surfaces; the cytotoxic effect of [Cu_2_(BPXD)]^4+^ complex is also assessed in vitro on the human fibroblast cell line.

## 2. Results and Discussion

### 2.1. Studies in Solution

The bisdien macrocycle BPXD was obtained with a well-established procedure, based on the formation of the [2+2] polyimine macrocycle, followed by reduction with NaBH_4_, as first described by Martell [16].

**Figure 1:**
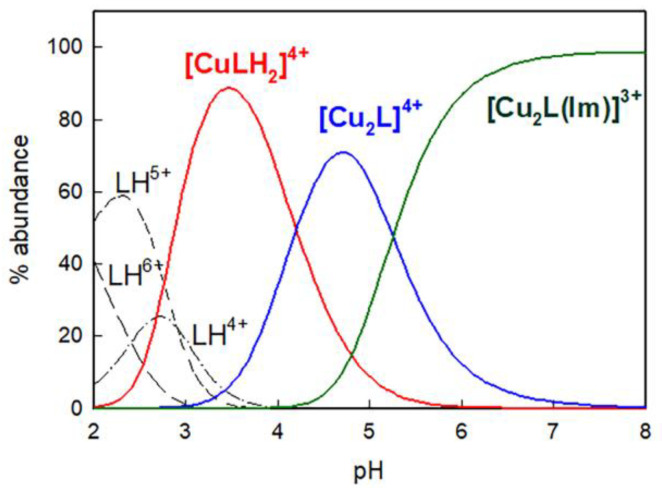
Distribution diagram obtained for aqueous solutions containing Cu(II), BPXD (indicated as L) and imidazole in 2:1:1 molar ratio ([BPXD] = [ImH] = 5 × 10^−4^ M, [Cu(CF_3_SO_3_)_2_] = 1 × 10^−3^ M in 0.05 M CF_3_SO_3_Na, T = 25 °C).

Potentiometric titrations in aqueous solution (0.05M CF_3_SO_3_Na, T = 25 °C) allowed us to determine the following: (i) the protonation constants for the free BPXD macrocycle, (ii) the equilibrium constants for BPXD in the presence of Cu(II) ions in a 1:2 BPXD:Cu(II) molar ratio, (iii) the complexation constants in the presence of imidazole (ImH) for a solution containing BPXD/Cu^2+^/ImH in 1:2:1 molar ratio. The stability constants for all equilibria are reported in Table 1. Figure 1 shows the distribution diagram of the species as a function of pH, calculated for an aqueous solution containing BPXD/Cu^2+^/ImH ([BPXD] = 5 × 10^−4^ M). The corresponding distribution diagram calculated in the absence of ImH is reported in the Supplementary Material (see Figure S1). As for the protonation equilibria of imidazole (ImH), the protonation constants shown in Table 1 were calculated in the same conditions as those employed for BPXD.

The association constants in Table 1 are in agreement with those already reported in literature [24], even though measured in different conditions of ionic strength, showing the complete (>90%) formation of [Cu_2_(BPXD)]^4+^ after pH 5, with deprotonation of water molecules coordinated to Cu(II) starting from pH 6 onward. Potentiometric titrations were stopped at pH 8, as above that pH value precipitation was observed. Quite curiously, in literature there are no stability constant data for inclusion of imidazolate bridging the two cations in [Cu_2_(BPXD)]^4+^. From our titrations and distribution diagram it is clear that in the presence of imidazole (ImH), from pH 4 onward, we can observe the formation of stable [Cu_2_(BPXD)(Im)]^3+^ complex, which is complete (>90%) above pH 6.1. This confirms the well-known, very high stability of the cascade complex containing imidazolate (Im^−^) as a bridging ligand for the two Cu(II) centres, which are held in the “correct” position by the hexaza macrocycle [20,21,22].

**Figure 2:**
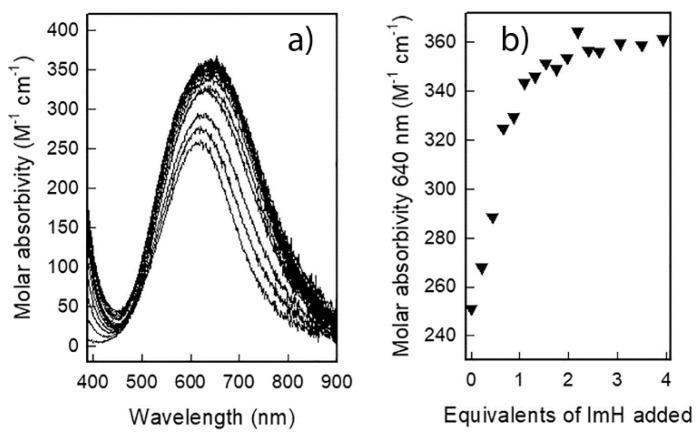
(**a**) Spectrophotometric titration of a [Cu_2_(BPXD)]^4+^ solution (5 × 10^−4^ M, buffered at pH = 6.0 with MES) with imidazole; (**b**) Titration profile showing molar absorptivity at 640 nm (black triangles) vs. added imidazole equivalents respect to [Cu_2_(BPXD)]^4+^.

The complexation of imidazolate by [Cu_2_(BPXD)]^4+^ can also be followed spectrophotometrically by titrating with imidazole a 5 × 10^−4^ M[Cu_2_(BPXD)]^4+^ solution in water buffered at pH 6, as shown in Figure 2. The typical d-d band usually observed in copper complexes in a triaminic compartment undergoes a red shift (from 620 to 640 nm) with broadening and slight increase of molar absorptivity, as already observed for imidazolate bridging in cascade complexes [22]. The profile of the titration obtained at 640 nm confirms the formation of a 1:1 adduct with imidazolate.

### 2.2. Bringing the Complex to Glass Surfaces

Synthesis of the imidazole containing grafting moiety **1** (see Scheme 1) was performed using the reaction between histamine and 3-(Isocyanato)propyltriethoxysilane to quantitatively yield 3-(Ureidoethylimidazole)propyltriethoxysilane, **1** [25].

**Figure 3:**
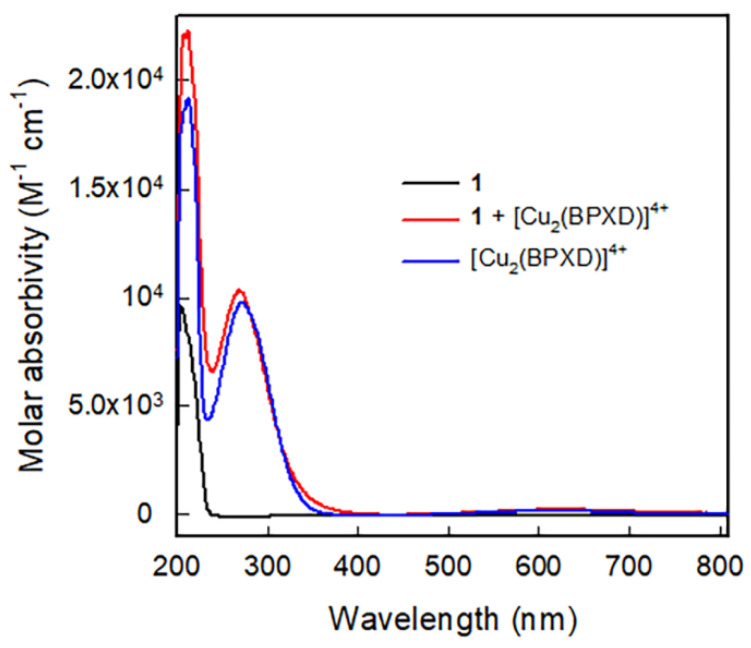
UV-vis spectra of: a 1.0 × 10^−4^ M solution of compound **1** (black line), same solution after addition of an equimolar amount of [Cu_2_(BPXD)]^4+^ (red line), a 1.0 × 10^−4^ EtOH solution of [Cu_2_(BPXD)]^4+^ (blue line).

As a first demonstration of the possibility of **1** to interact with [Cu_2_(BPXD)]^4^^+^, spectra were taken for a 1.0 × 10^−4^ EtOH solution of **1**, a 1.0 × 10^−4^ EtOH solution of [Cu_2_(BPXD)]^4+^ and a 1.0 × 10^−4^ EtOH solution of **1** in the presence of [Cu_2_(BPXD)]^4+^. Use of EtOH as solvent was needed to avoid hydrolysis of alcoxysilanes groups in **1**. Spectra are reported in Figure 3. These diluted conditions were necessary to measure LMCT bands of [Cu_2_(BPXD)]^4+^ placed in the UV range, which have high molar absorptivity, compared to the already cited d-d transitions.

The presence of absorption bands having high molar absorptivity, together with their calculation, is a fundamental prerequisite to evaluate the formation of a molecular monolayer on a bulk surface using spectrophotometry: thus, for this task, the observed d-d bands were unusable, as the molar absorptivity (about 3 × 10^2^ M^−1^ cm^−1^) was too low. As can be seen in Figure 3, molecule **1** had an absorption in the UV zone, between the lower range of spectrophotometer use and 230 nm, which could be attributed to imidazole moiety. Complex [Cu_2_(BPXD)]^4+^ had (in addition to the already cited d-d absorption at 620 nm), two strong bands at 210 nm and 270 nm, the second of which could be attributed to LMCT absorption from amines to the metal centres. In the presence of **1**, in addition to the already cited red-shift of the d-d band to 640 nm confirming complexation of imidazolate fragment of **1**, some slight changes of UV bands of [Cu_2_(BPXD)]^4+^ were observed. A similar experiment was repeated using imidazole instead of **1**, obtaining the same behavior (see Figure S2 in Supplementary Material), indicating undoubtedly that in EtOH solvent the [Cu_2_(BPXD)]^4+^ complex also binds to **1** by means of cascade interaction with the deprotonated imidazole moiety.

**Figure 4:**
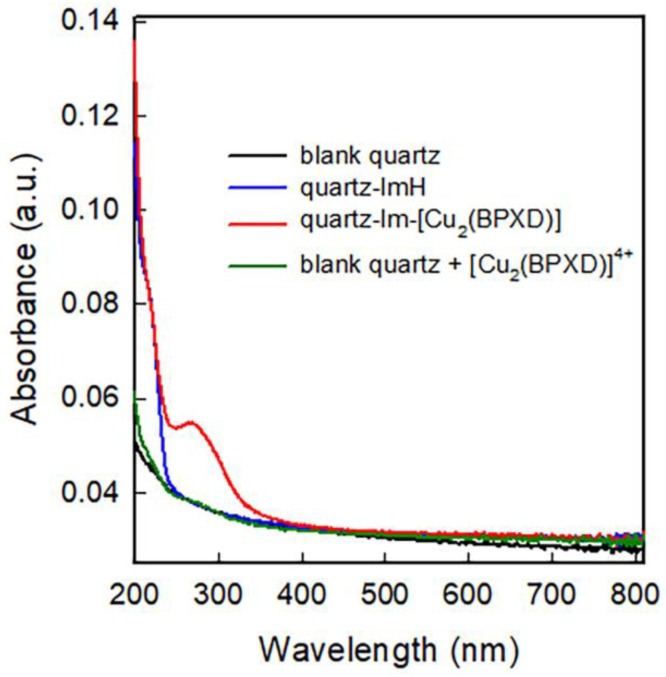
UV-vis spectra of a blank quartz (black line), a quartz-ImH sample (blue line), a quartz-Im-[Cu_2_(BPXD)] sample (red line) and of a blank quartz after immersion in a [Cu_2_(BPXD)](CF_3_SO_3_)_4_ solution (dark-green line).

Functionalization of glass or quartz surfaces with **1** can be obtained by simple immersion of properly activated glass or quartz samples in MeCN solutions of **1** for 4 h at 40 °C. Activation of glass and quartz samples was performed to maximize the number of silanolic functions present on the surfaces, and was done according to the prodedure described in Section 3.3. Success in silanization to yield glass-ImH and quartz-ImH samples was demonstrated by contact angle measurements, showing values which moved from less than 20°, for clean activated glass or quartz surfaces, to 40 (5)° after silanization. In the case of quartz-ImH, UV-vis spectra were taken and compared with those of a cleaned quartz sample, and the existence of an absorption zone placed around 220 nm was noticed, which confirmed that silanization had occurred, as reported in Figure 4 (blue line). Immersion of glass-ImH and quartz-ImH samples for 24 h in a 10^−3^ M EtOH solution of [Cu_2_(BPXD)](CF_3_SO_3_)_4_ to yield glass-Im-[Cu_2_(BPXD)] and quartz-Im-[Cu_2_(BPXD)] samples resulted in a further increase of surface contact angles to a value of 55 (5)°, imputable to the presence of phenyl spacers owing to BPXD. Spectra of quartz-Im-[Cu_2_(BPXD)] samples revealed absorption in the 200–300 nm region, with the presence of a neat band around 270 nm (red line in Figure 4), strictly resembling the LMCT band observed in solution (see Figure 3), and obviously assigned to the presence of [Cu_2_(BPXD)]^4+^ bound to imidazolate moieties grafted on quartz. When immersion in a EtOH solution of [Cu_2_(BPXD)](CF_3_SO_3_)_4_ was repeated using a clean quartz (in absence of grafting layer of **1**), negligible variations of the spectra of the clean quartz samples were obtained, indicating the marginal presence of a specific absorption [Cu_2_(BPXD)](CF_3_SO_3_)_4_ in the absence of a grafting layer (dark green line in Figure 4).

At this point, surface concentration values (n_s_) of bound complex could be calculated, starting from the extinction coefficient ε_270,sol_ at 270 nm found in a solution for [Cu_2_(BPXD)](CF_3_SO_3_)_4_ (as already noticed, the d–d band close to 640 nm was too weak to be observed when monolayers formed). A value close to 1.0 × 10^4^ M^−1^ cm^−1^ can be derived for ε_270,sol_ from the spectrum in Figure 3. We used a Lambert–Beer relation modified for monolayers of absorbing centres on surfaces to obtain the number of complex units brought to surfaces:

n_s_ (cm^−2^) = 6 × 10^20^A_270_,_surf_/2ε_270,sol_

where A_270,surf_ was the absorbance actually read at 270 nm for quartz-Im-[Cu_2_(BPXD)] samples. Factor 2 was used as the denominator because the quartz slides we used were functionalized on both sides. A mean value of n_s_ = 4.8 (2.1) × 10^14^ cm^−2^ was obtained upon spectra taken upon 10 quartz samples.

Surface density of [Cu_2_(BPXD)]^4+^ brought on the same quartz-Im-[Cu_2_(BPXD)] samples and on further synthesized glass-Im-[Cu_2_(BPXD)] samples could also be calculated by total Cu^2+^ quantitative analysis by ICP of solution obtained after dipping the samples in measured volumes of 0.1 M HNO_3_. A value of n_s_ = 5.8 (2) × 10^14^ cm^−2^ was found within these experiments, confirming the results of spectrophotometric investigations. These values were higher than what is usually observed for a monolayer, as the maximum density of Si–OH groups on a flat SiO_2_ surface is 5 × 10^14^ cm^−2^ [14], and suggest that some degree of vertical polymerization of **1** upon glass/quartz surfaces may have happened. Nevertheless, we can affirm that the proposed functionalization method allowed the grafting of a controlled, limited and reproducible quantity of complex on surfaces.

### 2.3. Study of Antimicrobial Action

As a first measure to evaluate antimicrobial effects, we decided to determine MIC (Minimum Inhibitory Concentration) and MBC/MFC (Minimum Bactericidal Concentration/Minimum Fungicidal Concentration) for [Cu_2_(BPXD)](CF_3_SO_3_)_4_ dissolved in solution and to compare it with the values found for free copper ions, which were investigated using Cu(CF_3_SO_3_)_2_ salt. In order to permit a proper comparison, we expressed these concentrations in mol/L values. *Escherichia coli* ATCC 10536, *Staphylococcus aureus* ATCC 6538, and *Candida albicans* ATCC 10,231 were used as representative strains of Gram-negative bacteria, Gram-positive bacteria and fungi, respectively. As can be seen in Table 2, the complex was much more active than Cu^2+^ ions as a microbicidal agent. The copper ions needed to reach at least 5 × 10^−3^ mol/L concentration to inhibit *S. aureus* growth, and even higher values for *E. coli*, while they were not found to be active for *C. albicans* at the highest concentrations investigated. On the contrary, inhibitory effects were found at much lower concentrations for [Cu_2_(BPXD)]^4+^ complex in solution (even considering that having a dinucleating complex the molarity of copper has to be doubled). MBC/MFC values were quite high, both for Cu^2+^ and [Cu_2_(BPXD)]^4+^, but again the complex in solution was found to be more active than the cations. Due to the partial insolubility of the ligand BPXD we were not able to discriminate if this effect was given by the whole complex or due to the ligand: in any case, as the ligand could not be loaded on functionalized surfaces without copper ions interacting with imidazole, we considered the measure of MIC and MBC/MFC of BPXD alone as poorly interesting.

Having assessed this effective antimicrobial action of [Cu_2_(BPXD)]^4+^, the microbicidal effect (ME) of the surfaces of glass-Im-[Cu_2_(BPXD)] was determined. ME was evaluated with an established procedure, in which an aqueous suspension of planktonic microbial cells was kept as a thin film placing a few microliters between a blank and a glass-Im-[Cu_2_(BPXD)] slide for 5 or 24 h (37 °C) [6].

NE, the number of surviving CFUs (colony forming units) in these conditions, was compared with NC, the number of CFUs in the control, which involved an identical setup but between two blank glass slides. At this point ME could be defined as ME = log(NC/NE). *E. coli*, *S. aureus*, and *C. albicans* were, again, used as representative strains. The results are summarized in Table 3.

Indeed, the glass-Im-[Cu_2_(BPXD)] surfaces showed a strikingly efficient ME after 24 h of contact for all of the three strains considered, while a lower, but still noticeable ME, was obtained for the shorter contact time of 5 h. It is important to stress the fact that a value of ME = 5, which is the maximum value, which was detectable with this experimental setup, corresponds to the complete elimination (99.999%) of tested microorganisms compared to the control.

It is also worth noting that in the ME experiments, the pH was buffered at physiological value, 7.4, and at this pH the stability of the inclusion complex between grafted imidazole (in the deprotonated form, imidazolate) and [Cu_2_(BPXD)]^4+^ was quite high, as was demonstrated with the solution studies and distribution diagrams. These data indicated that the complex was strongly bound to imidazolate in the conditions of the experiment and with negligible tendencies to dissociation. The calculation could be done considering that all complex grafted on the surface by cascade complexation with imidazolate was diluted in the volume V used for the ME experiment. We could, thus, calculate the maximum effective concentration (*M_eff_*) of the complex in this volume *V* (10 microliters), considering the value of surface concentration found previously *n_s_* (4.8 × 10^14^ cm^−2^). As in our experiments the slides were 2.1 × 2.6 cm, the calculated M_eff_ = n_s_S/N_a_V reached a value close to 5 × 10^−4^ M, the same used for the potentiometric titrations and distribution diagram calculation. In these conditions, thus, the whole of the complex [Cu_2_(BPXD)]^4+^ was expected to be firmly bound to imidazolate grafted on the surface, exactly as it was bound to imidazolate when in solution. Nevertheless, this stable complex is expected to dissociate with [Cu_2_(BPXD)]^4+^ getting progressively displaced when undergoing interaction with microorganism membranes which come in contact with the surfaces. The increase of the microbicidal action with contact time and the success in almost complete removal of microorganisms from surfaces, even with the low activity evidenced by high MIC and MBC/MFC values, can be explained as the result of accumulation of complexes inside cells. Similar behavior was already observed with sustained release of Ag^+^ from silver nanoparticles or Cu^2+^ from CuS nanoparticles grafted on glass [26], and is an essential feature. An antibacterial activity working for at least 24 h on planktonic bacteria is a requirement to impart to biomedical surfaces the ability to prevent the initial phase of biofilm growth: for example, bacterial adhesion to surfaces of a subcutaneous implant usually takes place in the first 12 h after the implantation [4].

### 2.4. Cytotoxicity Test

The cytotoxic effect of both Cu^2+^ and [Cu_2_(BPXD)]^4+^ solutions on the viability of human dermal fibroblasts (NHDF) was investigated; a Cu^2+^ solution was prepared in MilliQ water considering a Cu^2+^ concentration (2 × 10^−2^ M) equal to the one present in the [Cu_2_(BPXD)]^4+^ solutions, which were prepared by dilution of a stock 10^−2^ M concentration.

**Figure 5:**
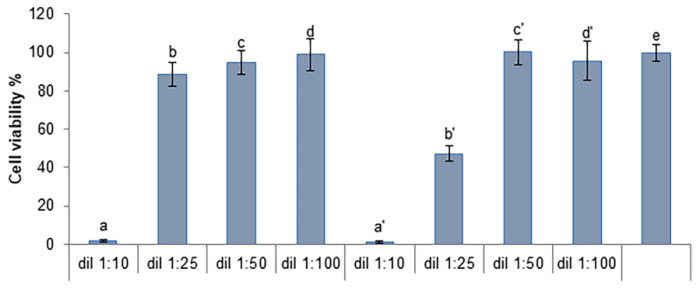
Cell viability, expressed as percentage of metabolically active cells, after treatment with the samples diluted in CM (1:10, 1:25, 1:50 and 1:100 (*v*/*v*)). a–d: [Cu_2_(BPXD)](CF_3_SO_3_)_4_; a’–d’: Cu(CF_3_SO_3_)_2_; CM (e) was used as reference (mean value ± s.d.; n= 6). ANOVA one-way; Sheffé test (*p* < 0.05): a vs. b–d, e; b vs. b’; a’ vs. b’–d’, e; b’ vs. c’, d’, e.

As reported in Figure 5, cell viability %after treatment with [Cu_2_(BPXD)]^4+^ was comparable with the one found using Cu^2+^ when concentrations did not exceed 2 × 10^−4^ M of complex (corresponding to a 4 × 10^−4^ M concentration in Cu^2+^): in both cases, these concentrations were not harmful to cells. When increasing the concentration up to 4 × 10^−4^ M, [Cu_2_(BPXD)]^4^^+^, still considered safe, there was a viability close to 90%, while free copper ions at 8 × 10^−4^ M entailed a reduction in cell viability to 45%. At 10^−3^ M concentration of [Cu_2_(BPXD)]^4+^ viability was reduced to zero and the same happened when using 2 × 10^−3^ M Cu(II) solutions.

When considering viability results and data coming from antimicrobial studies together, one can see that [Cu_2_(BPXD)]^4+^ can be considered safer and more active as an antimicrobial than free copper ions. [Cu_2_(BPXD)]^4+^ can be used up to 4 × 10^−4^ M providing a sensible antimicrobial action, as this value substantially equals the MIC value, at least for the two bacteria investigated. On the other hand, at these concentrations, copper ions are toxic to cells, but not active as an antimicrobial.

## 3. Materials and Methods

### 3.1. Materials and General Procedures

Unless otherwise stated, all reagents and solvents were supplied by Sigma-Aldrich and used as received. The synthesis and characterization of 3,6,9,16,19,22-hexaazatricyclo [22.2.2.2(11.14)]triaconta-1(26),11(12),13,24,27,29-hexaene (BPXD) and 3-(ureidoethylimidazole)propyltriethoxysilane (**1**) compounds are already reported in the literature [16,25]. The potentiometric titrations were made with a Radiometer TitraLab 90 titration system. Titration data were processed with the Hyperquad package [27] to determine the equilibrium constants.

UV-vis absorption spectra were recorded on a Cary 60 (Varian Ltd., Mulgrave, Victoria, Australia) spectrophotometer using a 1 cm path-length optical-glass cuvette or a proper sample holder for glass and quartz samples. Cover slides for microscopy ForLab 21 × 26 mm were purchased from Carlo Erba (Milan, Italy). Quartz slides (25 × 25 × 1 mm) were purchased from UQG-Optics (Cambridge, England).

### 3.2. Potentiometric Titrations

All potentiometric titrations were performed in aqueous solution, 0.05 M in CF_3_SO_3_Na, under nitrogen atmosphere using carbonate-free NaOH (T = 25 °C). In a typical titration experiment, a 5.0 × 10^−4^ M solution of BPXD (15 mL) was treated with an excess of a 1.0 M standard solution of CF_3_SO_3_H. Titrations were run by the addition of 10 μL portions of a standard 0.1 M solution of NaOH, collecting 80–100 points for each titration. Prior to each potentiometric titration, the standard electrochemical potential (E°) of the glass electrode was determined by a titration experiment according to the Gran method. The best fits of the potentiometric titration profiles for BPXD were obtained by assuming the presence of six protonated species at the equilibrium over the course of the potentiometric experiment. The calculated protonation constants (Table 1) allowed calculation of the species distribution diagram (as % abundance with respect to BPXD vs. pH). Potentiometric titrations were also performed in the same conditions on aqueous solutions containing either BPXD/Cu(CF_3_SO_3_)_2_ in 1:2 molar ratio or BPXD/Cu(CF_3_SO_3_)_2_/ImH in 1:2:1 molar ratio.

### 3.3. Glassware, Glass Slides and Quartz Slides Cleaning

Glassware was filled with aqua regia for 30 min, then filled and washed with bi-distilled water in an ultrasonic bath for 10 min. Water was discarded from the washing cycle with bi-distilled water/ultrasonic bath being repeated 2 more times. Finally, glassware was dried in an oven at 140 °C for 1h. Before reaction with silanes, glass and quartz slides were dipped for 30 min in a piranha solution (3:1 *v*/*v* 96% H_2_SO_4_/30% H_2_O_2_), then washed with bi-distilled water in an ultrasonic bath for 10 min. Water was discarded and the washing cycle with bi-distilled water/ultrasonic bath was repeated 2 more times. Finally, glassware was dried in an oven at 140 °C for 1h.

### 3.4. Preparation of Glass-ImH and Quartz-ImH Samples

A 0.10% solution of **1** in MeCN was used to fill a proper vessel, staining jars for microscopy able to bring 4 or 8 slides in a vertical position, in order to completely cover the activated glass or quartz samples. Vessels were placed in an oven at 40 °C for 4 h; after the reaction, the MeCN solution was discharged and the vessel containing the coated glasses was filled with fresh MeCN and sonicated for 3 min, and, after this, the MeCN was discharged. This washing procedure was repeated three times. After this, samples were blow-dried with N_2_.

### 3.5. Preparation of Glass-Im-[Cu_2_(BPXD)] and Quartz-Im-[Cu_2_(BPXD)] Samples

A solution containing 10^−3^ mol/L of [Cu_2_(BPXD)]^4+^ was prepared in EtOH and used to fill the desired vessel, in order to completely cover the of glass-ImH and quartz-ImH samples. Vessels were left at room temperature for 4 h to allow complexation to imidazolate grafted on glass.

After the reaction, the EtOH solution was discharged and the vessel containing coated glasses was filled with fresh EtOH and sonicated for 3 min, and, after this, EtOH was discharged. This washing procedure was repeated three times. After this, samples were blow-dried with N_2_.

### 3.6. Determination of Total Cu^2+^ by ICP

Each slide was treated in a 50 mL beaker with 3.0 mL bi-distilled water to which 0.180 mL 69 % HNO_3_ was added. The treated slides were left to react overnight and the solution was then analyzed using ICP, with a ICP- OES Optima 3300 DW Perkin Elmer instrument.

### 3.7. UV-VIS-NIR Absorption Spectra

Absorption spectra in solution were carried out in 1 cm glass cuvettes or in 1 mm quartz cuvettes using a Varian Cary 60 between 300 nm and 900 nm (glass) or 190–800 nm (quartz).

Absorption spectra of quartz-functionalized slides were carried out using a Varian Cary 60 spectrophotometer equipped with a solid sample holder, in the 190–800 nm range.

### 3.8. Antibacterial/Antifungal Activity Tests

The antibacterial and antifungal activity of compounds and functionalized surfaces was investigated against *Staphylococcus aureus* ATCC 6538 (Gram+), *Escherichia coli* ATCC 10,356 (Gram-) and *Candida albicans* ATCC 10231. The microorganisms were grown overnight in Tryptone Soya Broth (Oxoid; Basingstoke, Hampshire, England) and Potato Dextrose Broth (PDB) (DIFCO, Detroit, MI, USA) at 37 °C. Washed cells were resuspended in Dulbecco’s PBS and optical density (OD) was adjusted to 0.1, corresponding approximately to 1 × 10^8^ Colony Forming Units (CFU/mL) at 650 nm wavelength.

Evaluation of minimum inhibitory concentration (MIC) and minimum bactericidal-fungicidal concentration (MBC-MFC). All compounds, [Cu_2_(BPXD)]^4+^ and Cu(CF_3_SO_3_)_2_ salt were used, at different concentrations, in the evaluation of the antimicrobial activity against the reference strains. MICs and MBCs-MFCs were determined by a twofold serial broth dilution method in Iso-Sensitest broth (ISB, Oxoid, Basingstoke, UK) according to Clinical and Laboratory Standards Institute (CLSI; formerly NCCLS) procedures [28]. The starting inoculum was 1.0 × 10^8^ CFU/mL. The MIC was the lowest extract concentration inhibiting observable microbial growth after 24 h incubation at 37 °C. The MBC–MFC was the lowest concentration resulting in >99.9% reduction of the initial inoculum after 24 h incubation at 37 °C. All experiments were performed in triplicate [29].

Evaluation of microbicidal effect (ME). The antimicrobial activity of functionalized surfaces was determined against the reference strains. An amount of 10 μL of bacterial/fungal suspension was deposited on a standard glass slide (76 × 26 mm), then, the microbial suspension was covered with a glass-Im-[Cu_2_(BPXD)] glass slide (21 × 26 mm), forming a thin film between the slides that facilitated direct contact of the microorganisms with the active NP surface. The two assembled glasses were introduced in a Falcon test-tube (50 mL) containing 1 mL of PBS to maintain a damp environment. For each bacterial strain, two equivalent modified glasses were prepared; the slides were maintained in contact with the liquid films containing bacteria at room temperature for 5 and 24 h, respectively; for each time of contact an unmodified glass slide was treated in the same way as the control sample. After the periods of time of contact, 9 mL of PBS were introduced in each Falcon test-tube under a gentle shaking to detach the assembled glass slides. Bacterial suspensions were then grown in Tryptone Soya Agar (Oxoid; Basingstoke, Hampshire, UK) to count viable cells.

The decimal-log reduction rate, Microbicidal effect (ME), was calculated using the formula:

ME = log NC − log NE

(NC being the number of CFU/mL developed on the unmodified control glasses, and NE being the number of CFU/mL counted after exposure to modified glasses). The results expressed as ME represent the average of 3 equivalent determinations.

### 3.9. Cytotoxicity Test

For the experiments with Normal Human Dermal Fibroblasts (NHDF, PromoCell, Heidelberg, Germany), the materials hereafter reported were used. Dulbecco’s Modified Eagle’s Medium was purchased from Sigma Aldrich (Milan, Italy), while inactivated Fetal Bovine Serum (FBS) from Biowest (Nuaillé, France). Phosphate Buffer Solution (PBS), antibiotic/antimycotic solution (100×; stabilized with 10,000 units penicillin, 10 mg streptomycin, and 25 g amphotericin B per ml) and trypsin–EDTA solution was purchased from VWR (Radnor, PA, USA). Dimethyl sulfoxide (DMSO), MTT (3-(4,5-dimethylthiazol-2-yl)-2,5-diphenyltetrazolium bromide) and Trypan Blue solution were purchased from Sigma-Aldrich (Milan, Italy).

The cytotoxic effect of both Cu^2+^ and [Cu_2_(BPXD)]^4+^solutions on the viability of NHDF was investigated (please note: Cu^2+^ solution was prepared in MilliQ water, considering a Cu^2+^ concentration equal to that used for [Cu_2_(BPXD)]^4+^.

Cells were seeded on 96-well plates (3.5 × 10^4^ cells in 200 μL of complete culture medium (CM)/well) and incubated (37 °C and 5% CO_2_) for 24 h in order to reach semi-confluence. Samples, prepared in MilliQ water, were diluted 1:10, 1:25 and 1:50 (*v*/*v*) in complete culture medium (CM). An amount of 200 μL of each sample was put in contact for 24 h with cells; CM was used as reference. After incubation, an MTT assay was performed.

Briefly, samples and reference were removed from the 96-well plate and cell monolayers were washed with Phosphate Buffer Solution (PBS); subsequently, 50 μL of MTT 7.5 μM in 100 μL of DMEM without phenol red were added to each well and incubated for 3 h (37 °C and 5% CO_2_). Finally, 100 μL of DMSO was added to each well in order to promote the complete dissolution of formazan crystals, obtained from MTT dye reduction by mitochondrial dehydrogenases of living cells. The solution absorbance was measured by means of an iMark^®^ Microplate reader (Bio-Rad Laboratories S.r.l., Segrate Milano, Italy) at a wavelength of 570 nm and 690 nm (reference wavelength) after 60 s of mild shaking.

Results were expressed as % cell viability by normalizing the absorbance measured after contact with each sample with that measured for CM. Six replicates were performed for each sample.

## 4. Conclusions

To our knowledge, this is the first report in which a genuine supramolecular approach, which exploits coordinative interactions leading to formation of a cascade complex, is used for the realization of antimicrobial surfaces. Some more investigations are needed in order to deepen the comprehension of the antimicrobial action, and some effort has to be scheduled to find components suited to decreasing MIC and MBC/MFC, while, at the same time, lowering cytotoxicity to human cells. Nevertheless, we demonstrated how very low quantities of a copper complex brought to surfaces using supramolecular chemistry tools can lead to a high microbicidal effect: we, thus, believe this preliminary study will pave the way for a new generation of antimicrobial surfaces based on the power of metal–ligand interactions.

## Figures and Tables

**Table 1:** Global constants for the protonation and complexation equilibria studied in this work. The constants were determined through potentiometric titrations on BPXD (i.e., **L** in this table) in aqueous solution, 0.05 M CF_3_SO_3_Na, T = 25 °C. The protonation equilibria for ImH were investigated in the same conditions as for BPXD; the obtained results are consistent with the literature [23].

Equilibrium	logβ
L + H^+^ ↔ LH^+^	9.64 (3)
L + 2H^+^ ↔ LH_2_^2+^	18.22 (4)
L + 3H^+^ ↔ LH_3_^3+^	26.30 (4)
L + 4H^+^ ↔ LH_4_^4+^	33.23 (5)
L + 5H^+^ ↔ LH_5_^5+^	36.15 (5)
L + 6H^+^ ↔ LH_6_^6+^	38.0 (1)
L + 2Cu^2+^ ↔ [Cu_2_L]^4+^	26.17 (7)
L + Cu^2+^ + 2H^+^ ↔ [CuLH_2_]^4+^	30.87 (4)
L + 2Cu^2+^ + OH^−^ ↔ [Cu_2_L(OH)]^3+^	18.8 (1)
L + 2Cu^2+^ + 2OH^−^ ↔ [Cu_2_L(OH)_2_]^2+^	10.6 (1)
Im^−^ + H^+^ ↔ ImH	14.50 (1)
Im^−^ + 2H^+^ ↔ ImH_2_^+^	21.2 (1)
L + 2Cu^2+^ + Im^−^ ↔ [Cu_2_L(Im)]^3+^	40.7 (1)

**Table 2:** MIC and MBC/MFC values obtained for [Cu_2_(BPXD)](CF_3_SO_3_)_4_ complex compared with values obtained with Cu(CF_3_SO_3_)_2_ salt.

		*E. coli*	*S. aureus*	*C. albicans*
Cu(CF_3_SO_3_)_2_	MIC (mol/L)	0.010	5.0 × 10^−3^	>0.010
	MBC/MFC (mol/L)	0.010	0.010	>0.010
[Cu_2_(BPXD)](CF_3_SO_3_)_4_	MIC (mol/L)	4.0 × 10^−4^	3.0 × 10^−4^	7.0 × 10^−4^
	MBC/MFC (mol/L)	0.010	1.25 × 10^−3^	2.5 × 10^−3^

**Table 3:** Microbicidal effect (ME) of glass-Im-[Cu_2_(BPXD)] samples for different contact times.

	Microbicidal Effect (ME)		
	*E. coli*	*S. aureus*	*C. albicans*
Contact time			
5 h	3.8 (±0.8)	2.9 (±0.8)	1.2 (±0.7)
24 h	>5	5 (±1)	>5

## Data Availability

Not applicable.

## Supplementary Materials

The following supporting information can be downloaded at: https://www.mdpi.com/article/10.3390/molecules27175731/s1, Figure S1: distribution diagram obtained for solutions containing Cu(II) and BPXD 2:1 molar ratio; Figure S2: Uv-vis spectra of EtOH solution of imidazole, [Cu_2_(BPXD)]^4+^ and their 1:1 mix.

## Sample Availability

Samples of the compounds BPXD and **1** are available from the authors.

## Abbreviations

BPXD	3,6,9,16,19,22-hexaazatricyclo [22.2.2.2(11.14)]triaconta-1(26),11(12),13,24,27, 29-hexaene
CM	Culture Media
glass-ImH	Glass slides functionalized with grafted imidazole moieties
glass-Im-[Cu_2_(BPXD)]	Glass slides functionalized with imidazolate moieties and copper complexes
ICP	Inductively coupled plasma
ImH	Imidazole
Im^−^	Imidazolate
LMCT	Ligand to Metal Charge Transfer
MBC	Minimum Bactericidal Concentration
ME	Microbicidal Effect
*M_eff_*	Maximum Effective Concentration
MFC	Minimum Fungicidal Concentration
MIC	Minimum Inhibitory Concentration
quartz-ImH	Quartz slides functionalized with imidazole moieties
quartz-Im-[Cu_2_(BPXD)]	Quartz slides functionalized with imidazolate moieties and copper complexes
