# Supplementary material for: A Supramolecular Approach to Antimicrobial Surfaces
Source: Molecules. 2022 Sep 5;27(17):5731. doi: 10.3390/molecules27175731 (PMC9457607; doi:10.3390/molecules27175731)

SUPPLEMENTARY MATERIAL

A Supramolecular Approach to Antimicrobial Surfaces

Valentina Gazzola [1], Pietro Grisoli [2], Valeria Amendola [1], Giacomo Dacarro [1], Carlo Mangano [1], Piersandro Pallavicini [1], Antonio Poggi [1], Silvia Rossi [2], Barbara Vigani [2] and Angelo Taglietti [1,*]

[1] Department of Chemistry, University of Pavia, viale Torquato Taramelli 12, 27100 Pavia, Italy
[2] Department of Drug Sciences, University of Pavia, viale Torquato Taramelli 12, 27100 Pavia, Italy
* Correspondence: angelo.taglietti@unipv.it

Figure S1: distribution diagram obtained for solutions containing Cu(II) and BPXD in 2:1 molar ratio

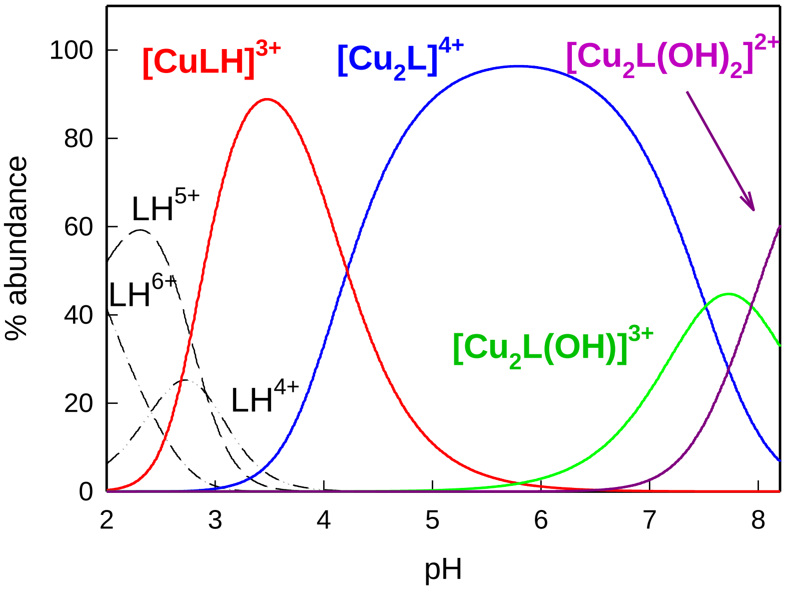

Figure S2: (up) Uv-vis spectra of: a 1.0x10^{-4} M solution of imidazole (black line) in EtOH, same solution after addition of an equimolar amount of $[Cu_2(BPXD)]^{4+}$ (red line), a 1.0x10^{-4} EtOH solution of $[Cu_2(BPXD)]^{4+}$ (blue line); (bottom) same spectra with emphasis on change of d-d band as a consequence of complezation of imidazolate as briding ligand between the two Cu^{2+} ions of $[Cu_2(BPXD)]^{4+}$

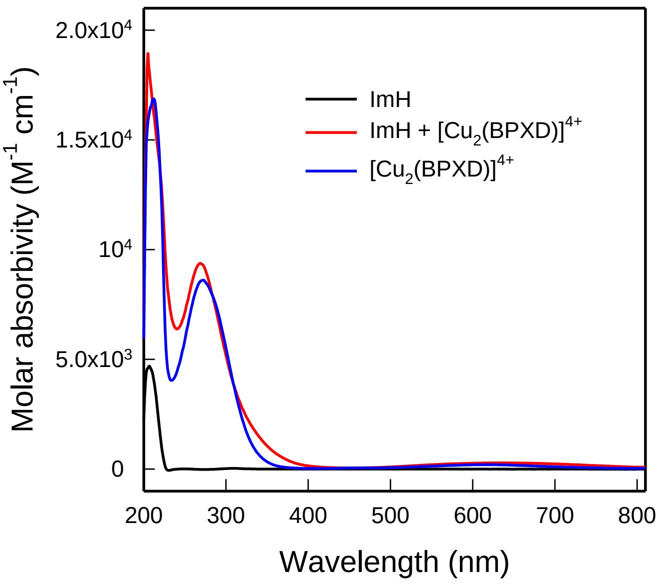

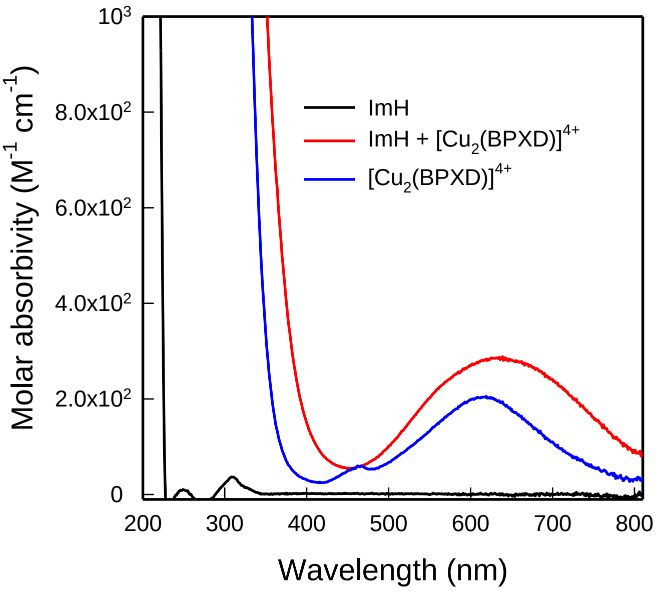

Supplement: Supplementary file 1 [file molecules-27-05731-s001.zip › molecules-1863334-supplementary.pdf]
